# The Tumor Necrosis Factor α (-308 A/G) Polymorphism Is Associated with Cystic Fibrosis in Mexican Patients

**DOI:** 10.1371/journal.pone.0090945

**Published:** 2014-03-06

**Authors:** Celia N. Sanchez-Dominguez, Miguel A. Reyes-Lopez, Adriana Bustamante, Ricardo M. Cerda-Flores, Maria del C. Villalobos-Torres, Hugo L. Gallardo-Blanco, Augusto Rojas-Martinez, Herminia G. Martinez-Rodriguez, Hugo A. Barrera-Saldaña, Rocio Ortiz-Lopez

**Affiliations:** 1 Departamento de Bioquimica y Medicina Molecular, Facultad de Medicina, Universidad Autonoma de Nuevo Leon, Monterrey, Nuevo Leon, Mexico; 2 Laboratorio de Medicina de Conservacion, Centro de Biotecnologia Genomica, Instituto Politecnico Nacional, Reynosa, Tamaulipas, Mexico; 3 Clinica de Fibrosis Quistica, Centro de Prevencion y Rehabilitacion de Enfermedades Pulmonares Cronicas (CEPREP), Facultad de Medicina y Hospital Universitario “Dr. Jose Eleuterio Gonzalez”, Universidad Autónoma de Nuevo Leon, Monterrey, Nuevo Leon, Mexico; 4 Facultad de Enfermeria, Universidad Autonoma de Nuevo Leon, Monterrey, Nuevo Leon, Mexico; 5 Departamento de Genetica, Facultad de Medicina, Universidad Autonoma de Nuevo Leon, Monterrey, Nuevo Leon, Mexico; 6 Centro de Investigacion y Desarrollo en Ciencias de la Salud, Universidad Autonoma de Nuevo Leon, Monterrey, Nuevo Leon, Mexico; University of Alabama-Birmingham, United States of America

## Abstract

Environmental and genetic factors may modify or contribute to the phenotypic differences observed in multigenic and monogenic diseases, such as cystic fibrosis (CF). An analysis of modifier genes can be helpful for estimating patient prognosis and directing preventive care. The aim of this study is to determine the association between seven genetic variants of four modifier genes and CF by comparing their corresponding allelic and genotypic frequencies in CF patients (n = 81) and control subjects (n = 104). Genetic variants of MBL2 exon 1 (A, B, C and D), the IL-8 promoter (−251 A/T), the TNFα promoter (TNF1/TNF2), and SERPINA1 (PI*Z and PI*S) were tested in CF patients and control subjects from northeastern Mexico by PCR-RFLP.

**Results:**

The TNF2 allele (*P = *0.012, OR 3.43, 95% CI 1.25–9.38) was significantly associated with CF under the dominant and additive models but was not associated with CF under the recessive model. This association remained statistically significant after adjusting for multiple tests using the Bonferroni correction (*P = *0.0482). The other tested variants and genotypes did not show any association with the disease.

**Conclusion:**

An analysis of seven genetic variants of four modifier genes showed that one variant, the TNF2 allele, appears to be significantly associated with CF in Mexican patients.

## Introduction

Gene-environment and gene-gene interactions play a role in the phenotypic expression of genetic diseases in individuals harboring the same genotype [1]. Cystic fibrosis (CF) has an estimated incidence of one in 3000 in the Caucasian population, although its frequency may vary in specific subgroups. A newborn screening study conducted in Mexico City revealed two CF-affected newborns among 7193 screened (1∶3597) participants, suggesting a high frequency of CF among Mexicans [2]. Approximately 1900 mutations and variants have been reported in the CF transmembrane conductance regulator (CFTR) gene, with ΔF508 being the most prevalent mutation (50%–60%, http://www.genet.sickkids.on.ca/app). CF primarily involves epithelial cells in the respiratory tract, intestine, pancreas, bladder, and sweat glands; respiratory failure, however, is the major cause of death in CF patients [3].

Variants in genes that are involved in the inflammatory response have been studied in CF patients based on their potential effects on inflammation and host defense mechanisms. The mannose binding lectin (MBL2) gene encodes a serum acute-phase protein secreted by the liver, resembling the complement component C1q, that leads to opsonization and activation of the complement system through the classical pathway [4]. The serum concentration and complement-triggering activity of MBL depend on single-base mutations in the MBL2 gene [5]–[7]. These mutations may increase the susceptibility of carriers to colonization by bacterial and viral pathogens [8]. The best known genetic variants in exon 1 of the MBL2 gene are Gly54Asp (the B allele, rs1800450), Gly57Glu (the C allele, rs1800451) and Arg52Cys (the D allele, rs5030737), which are together referred to as the O allele. The interleukin 8 (IL-8) gene codes for a member of the CXC chemokine family and is mainly involved in the initiation and amplification of acute inflammatory reactions [9]. IL-8 is produced by a wide range of cell types, such as monocytes, macrophages, and fibroblasts; it primarily mediates the activation and migration of neutrophils from peripheral blood into pathogen-infected tissue, initiating and amplifying inflammatory processes [10]. A polymorphism in position −251 of the IL-8 gene (rs4073) is associated with increased IL-8 expression [11]–[12]. The tumor necrosis factor alpha (TNFα) gene expresses a multifunctional pro-inflammatory cytokine secreted in response to numerous specific stimuli, such as lipopolysaccharides. This molecule induces the release of cytokines IL-6 and IL-8 and increases airway mucus production [13]–[14]. The −308 A TNFα promoter polymorphism (TNF2, rs1800629) has been associated with increased TNFα transcription activity relative to the normal TNF1 allele (−308 G) [15]–[17]. The Alpha-1-antitrypsin (AAT, SERPINA1) gene codes for an acute-phase serine protease glycoprotein that limits tissue self-damage during the inflammatory immune response. AAT deficiency, caused by the S (p.E264V, rs17580) and Z (p.E342K, rs28929474) alleles in the SERPINA1 gene, may induce liver and pulmonary disease [18]. Severe AAT deficiency is a co-dominant autosomal hereditary disorder that clinically resembles early onset pulmonary emphysema, particularly in smokers [19].

In our Cystic fibrosis clinic, we have a broad range of severity of the CF disease, even with patients carrying the same genotype. No reports on variations in modifier genes in Mexican CF patients have been previously published. Our goal was to explore polymorphisms in genes related to host defense in healthy controls and CF patients from northeastern Mexico to find differences in allelic distribution between both groups. In this study, we report the genotype and allele frequencies of seven genetic variants in four previously reported CF modifier genes: MBL, IL-8, TNFα and AAT.

## Materials and Methods

### Biological Samples

The study was approved by the Research and Ethics Committee of the Universidad Autonoma de Nuevo Leon University Hospital (Registry number BI09-003). After signing written informed consent, blood samples were drawn from 81 CF patients attending the Chronic Lung Disease Prevention and Rehabilitation Center (CEPREP, in Spanish) and from control subjects recruited from the University Hospital and School of Medicine (Universidad Autonoma de Nuevo Leon). Also we collected blood samples from 104 control subjects that met the following inclusion criteria: they agreed to informed consent, they were born in northeastern Mexico (the states of Nuevo Leon, Tamaulipas, Coahuila, and San Luis Potosi), and they belonged to a family with at least three ascending Mexican generations. Genomic DNA was isolated from peripheral venous blood using the phenol–chloroform method, precipitated in ethanol, and finally suspended in Tris-EDTA (pH 7.8).

### Screening for the CFTR Gene Mutations

Mutation screening was performed according to the availability of resources and kits along the time. For previously screened CF patients: direct detection of ΔF508 mutation, and Roche ASO16 and 27 mutations kits, for new CF patients: direct detection of ΔF508 mutation, INNOLiPA CFTR36 probe kit and Exon 11-specific PCR and sequencing. Short descriptions of the methodologies are presented below.

PCR and electrophoresis to detect the ΔF508 mutation: PCR product was analyzed in polyachrylamide gels and the diagnosis was established comparing to molecular marker and DNAs of previously ΔF508 diagnosed patients and control subjects (98 bp band for normal allele or 95 bp band for ΔF508 mutation) [20].

Roche ASO16 and 27 kits (Roche Molecular Systems, Alameda CA, USA) or the INNOLiPA CFTR36 probe kit (Innogenetics, Ghent, Belgium). Methodology consisted of multiplex PCR reactions with biotinylated primers. After verifying the amplification in an agarose gel of 2%, the products were hybridized to membrane bound probes. A positive result was expressed as the appearance of a purple band. Both kits detected normal and mutated versions to report homozygous or heterozygous status for the CF patients. Complete list of mutations is shown in Table 1.

**Table 1:** Mutations included in the kits used for the molecular diagnosis of CF patients.

Kit	Detected mutations
ASO16 Roche	DF508, G542X, G551D, R553X, W1282X, N1303K, R117H, 621+1G >T, R334W, R347P, A455E, DI507, 1717-1G >A, S549N, R560T, 3849+10Kb C >T
ASO26 Roche	DF508, G542X, G551D, R553X, W1282X, N1303K, R117H, 621+1G >T, R334W, R347P, A455E, DI507, 1717-1G >A, R560T, 3849+10KbC>T, G85E, 2307insA, G480C, A559T, R1162X, 3659delC, S1255X, R347H, 2789+5G>A, 405+3 A>C, 3120+1G>A
INNOLiPA CFTR36 Innogenetics	F508del, G542X, G551D, R553X, W1282X, N1303K, R117H, 621+1G>T, R334W, R347P, A455E, I507del, 1717-1G>A, R560T, 3849+10KbC>T, G85E, R1162X, 3659delC, 2789+5G>A, 3120+1G>A, 711+1G>T, 3905insT, S1251N, Q552X, I148T, 1898+1G>A, 394delTT, 1078delT, 2183AA>G, 2184delA, E60X, 2143delT, 711+5G>A, 3199del6, 3272-26A>G, dele2,3

Exon 11- specific PCR and sequencing. Exon 11 PCR fragment was amplified and sequenced in one patient with absence of hybridization of PCR product on the normal and mutated versions of the G551D probe in the CFTR36 probe kit.

### Modifier Genes Analysis

**Figure 1:**
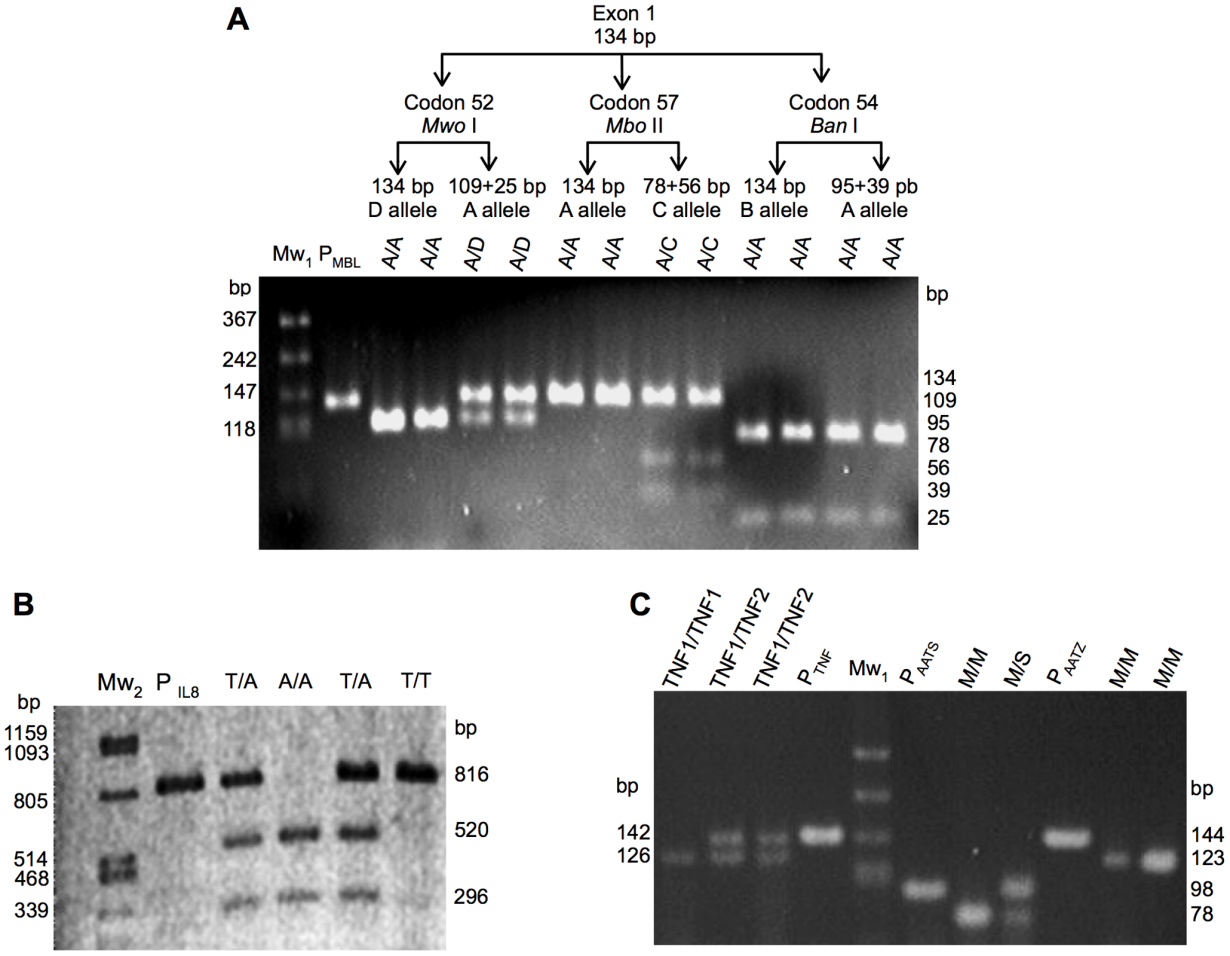
PCR-RFLP for the modifier genes analysis. 1A: the 134 bp PCR product from exon 1 of the MBL1 gene was digested with *Mwo* I, *Ban* I and *Mbo* II for detection of polymorphisms in the 52, 54 and 57 codons. 1B: the 816 bp PCR product from promoter region of the IL-8 gene was digested with *Mfe* I for detection of the −251 polymorphism. 1C: the 142 bp PCR product from promoter region of the TNFα gene was digested with *Nco* I for detection of the −308 polymorphism (TNF2); the 98 bp PCR product from the SERPINA1 gene was digested with *Taq* I^α^ for detection of the S genetic variant; the 144 bp PCR product from the SERPINA1 gene was digested with *Taq* I^α^ enzyme for detection of the Z genetic variant. Mw1 is the molecular marker pBs+*Msp* I, Mw2 is the molecular marker λ+*Pst* I. P_MBL_, P_IL8_, P_TNF_, P_AATS_ and P_AATZ_ are undigested PCR products. The Z allele was not detected.

DNA from CF patients and control subjects was tested for the Gly54Asp (B allele, rs1800450), Gly57Glu (C allele, rs1800451), Arg52Cys (D allele, rs5030737), and A (wild type) alleles in exon 1 of the MBL2 gene; the −251 T/A (rs4073) allele of the IL-8 gene; the −308 G/A (TNF1/2, rs1800629) alleles of the TNFα gene; and the PI*S Glu264Val (rs17580), PI*Z Glu342Lys (rs28929474), and PI*M (wild type) alleles of the SERPINA1 gene. PCR-RFLP protocols were adapted from previously reported methods. More details are explained in Table 2 and Figure 1 [21]–[24].

**Table 2:** Modifier genes analysis by PCR-RFLP adapted from previously published techniques [21]–[24].

Gene	Polymorphism	Primers	Enzyme	Mutant allele, bp	Normal allele, bp
MBL	Arg52Cys (D)	F: CAT CAA CGG CTT CCC AGG GCA AGA TGG G	*Mwo* I	134	109+25
	Gly54Asp (B)	R: GTC TCC TCA TAT CCC CAG GC	*Ban* I	134	95+39
	Gly57Glu (C)		*Mbo* II	78+56	134
IL-8	−251 T/A	F: GAT TCT GCT CTT ATG CCT CCA	*Mfe* I	816	520+296
		R: CCC AAG CTT GTG TGC TCT GCT GTC			
TNFα	−308 G/A	F: GGG ACA CAC AAG CAT CAA GG	*Nco* I	142	126+16
		R: AAT AGG TTT TGA GGG CCA TG			
AAT	PI*S Glu264Val	F: GAG GGG AAA CTA CAG CAC CTC G	*Taq* I^α^	98	78+20
		R: ACC CTC AGG TTG GGG AAT CAC C			
	PI*Z Glu342Lys	F: TAA GGC TGT GCT GAC CAT CGT C	*Taq* I^α^	144	123+21
		R: GGA GAC TTG GTA TTT TGT TCA ATC			

### Statistical Analysis

The SNP & Variation Suite (SVS) 7 (Golden Helix Inc., Bozeman, MA, USA) software program was used to perform all statistical analyses. The association between the tested genotypes and CF was analyzed by correlation/trend and chi-squared (χ^2^) tests under three different models (dominant, recessive, and additive) and was confirmed with the Bonferroni correction to detect the false discovery rate. Odds ratios were estimated within 95% confidence intervals. Values of *P*<0.05 were considered statistically significant. The Hardy–Weinberg Equilibrium (HWE) *P*-values were assessed using a chi-square test.

## Results

Eighty-one CF patients and 104 control subjects were recruited for this study. Genotype frequencies for the CFTR gene are described in Table 3. A complete genotype characterization was achieved in 55.6% (n = 45) of the CF patients; in 39.5% (n = 32) of the CF patients, only one mutation was identified, and in 4.9% (n = 4) of the CF patients, both mutations remained undetected. The most prevalent genotypes were ΔF508/other (46.9%, n = 38) and ΔF508/ΔF508 (35.8%, n = 29). The overall frequency of the ΔF508 allele among CF patients was 59.3%. Ten additional mutations were detected: G542X (4.9%), S549N (3.1%), 2789+5G>A (2.5%), 3849+10 kb (1.9%), G85E, R1162X, I148T, R334W, ΔI507, and L206W (0.6% each one). Mutations in the CFTR gene were not detected in 24.7% of the total CFTR alleles.

**Table 3:** CFTR genotype frequencies from 81 Mexican CF patients.

Genotype	N	%
ΔF508/ΔF508	29	35.8
ΔF508/X	26	32.1
ΔF508/G542X	5	6.2
ΔF508/3849+10 kb	3	3.7
S549N/S549N	2	2.5
G542X/X	2	2.5
ΔF508/S549N	1	1.2
ΔF508/L206W	1	1.2
ΔF508/2789+5G>A	1	1.2
ΔF508/G85E	1	1.2
2789+5G>A/2789+5G>A	1	1.2
G542X/R1162X	1	1.2
2789+5G>A/X	1	1.2
ΔI507/X	1	1.2
I148T/X	1	1.2
R334W/X	1	1.2
X/X	4	4.9
Total	81	100

X: unknown allele.

The PCR-RFLP patterns of the seven genetic variants in the four modifier genes are shown in the Figure 1. The Z allele, and the homozygote TNF2 and AATS genotypes were not found. The genotype frequencies of modifier genes in CF patients and control subjects are shown in Table 4. The B, C, and D alleles of the MBL2 gene were grouped together and reported as the O allele. The polymorphisms were in Hardy-Weinberg equilibrium. The frequencies of the mutant alleles for CF patients and controls were: the MBL-O allele 0.231 and 0.233, the IL-8 -251T allele 0.576 and 0.569, AATS allele 0.012 and 0.014, and TNF2 allele 0.087 and 0.029, respectively. The TNF2 allele (*P = *0.012, Odds Ratio (OR) 3.43, 95% CI 1.25–9.38) was significantly associated with CF patients using the dominant model. This association remains statistically significant after adjusting for multiple testing using the Bonferroni correction (*P* = 0.0482). The association value and the OR of the TNF2 allele were statistically significant when assessed using the additive model (Dd vs. dd) but not the recessive model (Table 5). The other genetic variants tested did not show any association.

**Table 4:** Modifier gene genotype frequencies (%) in CF patients and control subjects; OR, Hardy–Weinberg Equilibrium (HWE) and results of the association test with Dominant Model *P*-values.

Gene	Genotypes	Genotype Frequency inCF Patients	HWE P inCF Patients	Genotype Frequency inControls	HWE P inControls	OR (95% CI)Dominant Model	P-ValueDominant Model	
		N (%)		N (%)			X^2^	X^2^ Bonf. P
MBL2	AA	46 (57.5)	0.4210	63 (61.2)	0.1843	A 1.01 (0.62–1.65)	0.6163	1.0000
	AO	31 (38.8)		32 (31.1)		O 0.99 (0.61–1.61)		
	OO	3 (3.8)		8 (7.8)				
IL-8	AA	13 (16.5)	0.5786	19 (18.6)	0.9937	T 1.03 (0.68.1.57)	0.9194	1.0000
	AT	41 (51.9)		50 (49.0)		A 0.97 (0.64–1.48)		
	TT	25 (31.6)		33 (32.4)				
TNFα	TNF1/TNF1	66 (82.5)	0.3911	97 (94.2)	0.7608	aTNF1 0.30 (0.11–0.80)	0.0120	0.0482
	TNF1/TNF2	14 (17.5)		6 (5.8)		bTNF2 3.43 (1.25–9.38)		
	TNF2/TNF2	0 (0.0)		0 (0)				
AAT	MM	79 (97.5)	0.9104	101 (97.1)	0.8814	M 1.17 (0.19–7.01)	0.8627	1.0000
	MS	2 (2.5)		3 (2.9)		S 0.85 (0.14–5.17)		
	M/Z, S/Z, S/S, Z/Z	0 (0)		0 (0)				

CF, cystic fibrosis; OR, Odds Ratio; CI, confidence interval.

a TNF1 −308 G: (dd) vs. (DD, Dd),

b TNF2 −308 A: (DD, Dd) vs. (dd).

HW-P: *P*-value for the Hardy-Weinberg equilibrium.

X^2^ Bonf. P = Chi squared test with a Bonferroni-corrected *P*-value.

**Table 5:** TNF1/TNF2 association values using dominant, additive and recessive models.

Model	TNFα −308 G/−308 A OR^1^ (95% CI)	*P*-value	
Dominant	TNF1 = 0.29 (0.11–0.79)	0.012^2^	0.048^3^
	TNF2 = 3.43 (1.25–9.38)		
Additive	TNF1 = NA	0.012^4^	0.049^3^
	TNF2 = 3.429 (1.25–9.38)		
Recessive	NA	NA	NA

1 odds ratio.

2 Chi-Squared P.

3 Bonf. P.

4 Correl/Trend P.

## Discussion

In the present study, the ΔF508 mutation accounted for 59.3% of the mutated CFTR alleles, a frequency that resembles the mutation frequencies of European Mediterranean countries. In this regard, this work may be comparable with modifier genes studies performed in those countries. Two previous CFTR mutation reports in the Mexican population showed ΔF508 frequencies of 40.72% [25], 45% [26] and 44.6% [27]. Differences could be explained by the clinical criteria, geographic origin and the analytical methods available at that time. The Spanish federation reported a frequency of 45%. Previous studies in the Hispanic population reported a ΔF508 allele frequency ranging from 29 to 46% [28]–[30]. The frequencies of the G542X, R1162X and R334W alleles reflect the Spanish heritage in the Mexican population, but the S549N and 2789+5 G>A alleles are not among the most frequent in Spain [31]–[32]. In this study we previously detected homozygote S549N and ΔF508/S549N genotypes with the ASO16 kit. A second patient was detected by exon 11 direct sequencing because she presented an abnormal pattern of the INNOLiPA CFTR36 kit with the normal and mutant G551D probes. This kit along with the ASO27 excluded the S549N mutation from the mutation panel, hindering the CFTR molecular diagnosis in our population. CFTR gene sequencing should be performed for new or rare CFTR mutations in Mexican population as S549N, and those mutations obtained from central Mexico patients (P750L, 846delT, 4160insGGGG and 297–1 G>A) [25], as well as for those mutations that remained undetected. It is necessary to establish an adequate diagnosis strategy based in Mexican genetic profile, considering that available commercial kits are designed mainly for Caucasian mutations profile.

In northeastern Mexico, medical care for CF patients is offered at the Cystic Fibrosis Clinic of the CEPREP (http://www.ceprep.edu.mx). Since 1987, approximately 200 patients have been diagnosed with CF based on clinical and molecular analyses. Once the diagnosis of CF is established, the rate of adherence to medical treatment and long-term medical monitoring is low, making investigations into the genetic and environmental factors that influence the outcome in CF Mexican patients difficult.

In this study, we analyzed seven variants in four modifier genes previously reported in CF patients and we found an association between CF and the TNF2 allele.

The proinflammatory role of the TNF2 allele has been demonstrated in B cell line cultures, where the TNF2 allele was more potent transcriptional activator compared to the normal TNF1 [17]. TNF2 allele has been implicated as a potent immunomediator and pro-inflammatory cytokine in the pathogenesis of several human diseases, including pulmonary diseases as CF and asthma. Patients with genotypes related to higher TNFα production had increased frequency of asthma [33]. In Mexican population TNF2 allele was found in 6.0% of asthmatic compared to 2.9% of the controls [34]. These results are similar that those we found in our study (2.9% for controls and 8.9% for CF patients). By the other hand, recent studies in Mexican population reported a higher frequency of the TNF2 allele in healthy unrelated Mexican individuals (7.3%) [35]. Another report related TNF2 allele to breast cancer in Mexican patients compared to healthy women (7.5 and 24.5% respectively) [36]. Differences in TNF1 allele frequency in control subjects could be explained by sample size, methodology and characteristics of the studied group. Our study included population from Northeastern Mexico, while the other studies had different criteria as gender, living in Mexico City or been born in Mexico.

Previous studies in CF patients have shown that TNF2 is associated with a lower percentage of predicted forced expiratory volume in one second (FEV_1_) and weight z scores [37]. In Mexican population, the TNF2 allele has been associated with rheumatoid arthritis [38], geriatric lipid profile [39] and spondyloarthritis [40]. This variant has also been reported to be associated with obesity and asthma [41]–[42]. The high frequency of the TNF2 allele in Mexican CF patients could suggest a heterozygote advantage. In Colombia, an inverse association between the TNF polymorphism and autoimmunity and TB has been reported; this association suggests the existence of a heterozygote advantage and is consistent with the hypothesis that autoimmune diseases are a consequence of natural selection for enhanced TB resistance [43]–[44].

MBL2, IL-8 and AAT did not show an association with the CF genotype. MBL2 had previously shown associations with different disturbances in the lung function, infection risk, and survival of CF patients [6], [45]–[48]. In asthma, MBL has been associated with *Chlamydia pneumoniae*–specific IgG and a greater risk of developing asthma, especially in children with chronic or recurrent infection [49]. MBL levels in asthmatic children positively correlate with peripheral blood eosinophils [50]. MBL therapy may be useful in MBL-deficient patients; it may reduce the susceptibility to or enhance the recovery from bacterial infection or modify the natural history of the disease [51]–[53]. The IL-8 −251 polymorphism has been associated with CF lung disease severity and the differential expression of IL-8, suggesting that the IL-8 variant modifies CF lung disease severity [54]. The −251 variant has been associated with asthma, infection by respiratory syncytial virus, and chronic obstructive pulmonary disease (COPD) [10], [55]–[56]. Finally, despite the association between AAT deficiency and COPD, studies of AAT variants and infection in CF patients have been inconclusive [57]–[60]. The incidence of AAT deficiency for all five phenotypic classes of the Pi*S and Pi*Z deficiency alleles is 1 in 9.8 for Canada and 1 in 11.3 for the United States. However, a previous report from our group showed very low allele frequencies of Pi*S and Pi*Z variants in a Mexican population (1.5% and 0%, respectively) [61].

In summary, the frequencies of genetic variants in the MBL2, IL-8, and AAT genes of CF patients did not show significant differences when compared to control subjects, but the TNF2 allele was significantly associated with CF patients. More studies are needed to identify the role of inflammatory mediators in the pathophysiology of CF, as emphasized in previous studies. This information is relevant because clinical trials of drugs targeting TNFα activity [62] have shown outstanding efficacy in treating chronic inflammatory diseases.
